# Prospective randomized comparative study of use of PLMA and ET tube for airway management in children under general anaesthesia

**DOI:** 10.4103/0019-5049.63643

**Published:** 2010

**Authors:** Mamta G Patel, VN Swadia, Geetika Bansal

**Affiliations:** Department of Anesthesiology, Medical College and S.S.G. Hospital, Jail road, Vadodara 390001, Gujarat, India

**Keywords:** Anaesthesia: general anaesthesia, equipment: proseal laryngeal mask airway (plma), endotracheal tube (ett), patients: children

## Abstract

ProSeal^TM^ Laryngeal Mask Airway (PLMA) for children had been introduced in 2004, by Dr. Archie Brain. It has, in addition to Classic Laryngeal Mask Airway (CLMA), a drainage tube for providing a bypass channel for gastric contents to prevent regurgitation and pulmonary aspiration. A randomized prospective study was performed comprising of 60 ASA – I/II patients, between the age groups of 3 and 10 years, of either sex. All the patients were premedicated with oral Midazolam and Glycopyrollate. General anaesthesia with caudal epidural analgesia was given in all the cases. Inhalation with 8% Sevoflurane was used as a sole induction agent in all the patients. They were randomly divided into two groups. PLMA was inserted in patients of Group P and Endotracheal Tube (ETT) in patients of Group I. In all cases, after PLMA / ETT insertion; caudal epidural analgesia was given and general anaesthesia (GA) using Sevoflurane was provided for maintenance of anaesthesia. Muscle relaxant was not used in our study. We studied parameters such as number of attempts, ease of insertion and conditions during insertion, haemodynamic parameters, changes in SpO_2_, EtCO_2_, gastric insufflation, regurgitation, pulmonary aspiration, postoperative airway complications and so on. We found that insertion of PLMA as well as ETT was performed in the first attempt in all the patients. Ease of insertion and conditions during insertion were comparable in both the groups. Changes in SpO_2_ and EtCO_2_ were comparable. However, highly significant changes in haemodynamic parameters were observed in the ETT group. Complications such as sore throat (13.33% cases), coughing (40% cases), vomiting (3.33% cases) and hypoxia (3.33% cases) were observed in the ETT group. No gastric insufflation or regurgitation was noted in our study. Thus, we concluded that PLMA could be used as an effective and safe airway device in children compared to ETT undergoing general anaesthesia.

## INTRODUCTION

Laryngeal mask airway developed by Dr. Archie Brain is relatively non-invasive in comparison to endotracheal intubation and causes minimal disturbances in the cardiovascular and respiratory systems. It has a lesser risk of airway injury during the perioperative period.[1‐3]

The Proseal Laryngeal Mask Airway (PLMA) provides a more effective airway seal; it can be used safely for Positive Pressure Ventilation (PPV). The second drainage tube protects the patient from aspiration of gastric contents and prevents aspiration.

Proseal LMA offers as much protection as endotracheal intubation in children and avoids the risk associated with laryngoscopy and intubation, as observed in various scientific studies.[4‐7]

**Figure 1:**
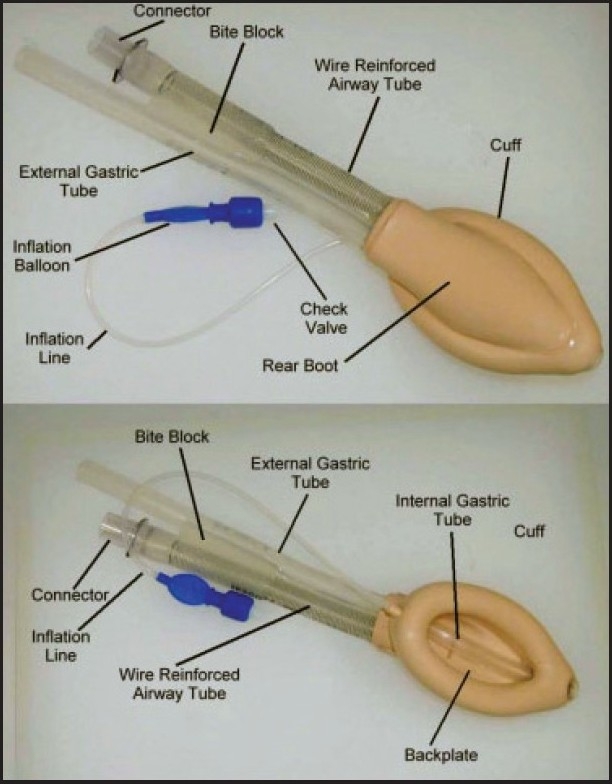
Photograph of paediatric LMA (PPLMA)

The paediatric PLMA, which has recently been introduced, in 2004, is not the scaled down version of the adult size,[5,8] as it lacks the dorsal cuff and has a relatively large drainage tube. Figure 1 shows the paediatric Proseal LMA (PPLMA).

PLMA is easy to use and causes less haemodynamic response during insertion and removal.[2,4,6,9,10] It has fewer complications such as coughing, sore throat, vomiting, laryngospasm and bronchospasm, in comparison to laryngoscopy and endotracheal intubation.[11]

We decided to undertake a study of comparison between insertion and removal of PLMA and ETT, using Sevoflurane as a sole induction agent, without using any muscle relaxant, in paediatric patients posted for lower abdominal surgeries.

## METHODS

After approval from the ethical committee, and written informed consent from the parents, a total of 60 children of ASA grade I/II and age between 3 and 10 years, weighing between 5 and 25 kg, undergoing elective lower abdominal surgical procedures such as circumcision, congenital hernia, congenital hydrocele, hypospadias, colostomy closure, skin grafting and appendicectomy were enrolled in our study [Table 1].

**Table 1:** Demographic data

Demographic data:				
Variables		Group P	Group I	*P* value
Age (years)		5.466 ± 1.870	5.233 ± 1.832	> 0.05
Weight (kg)		14.85 ± 2.927	13.466 ± 2.944	> 0.05
Sex	M	27	27	> 0.05
	F	3	3	> 0.05

ASA grade:				
ASA grade	Group P	Group I	*P* value

I		28	27	> 0.05
II		2	3	> 0.05

Patients with an anticipated difficult airway, with ASA grade III/IV, recent history of URTI and full stomach were excluded from our study.

In this prospective comparative randomized study, all the patients were randomly divided into two groups of 30 each. In our study, randomization was done by V.N.S. using the simple envelope method and the observations were made by M.G.P.

Group P: PLMA was inserted (30 patients)

Group I: Endotracheal tube was inserted (30 patients)

All the procedures such as PLMA insertion and intubation were performed by an anaesthesiologist who was experienced and trained in various types of LMA for three years.

After confirming nil oral status, all the patients were premedicated orally with Midazolam 0.5 mg/kg and Glycopyrollate 20 *μ*/kg, 30 minutes before induction of anaesthesia. EMLA cream was also applied at the dorsum of the hand 30 minutes before surgery. Baseline vital sign parameters were monitored in the form of pulse rate, blood pressure (NIBP), SpO_2_ and EtCO_2_.

General anaesthesia was induced with inhalation of Sevoflurane (8% concentration) with N_2_O and O_2_ (50:50). Loss of eyelash reflex was taken as an end-point of induction. Induction Time — time taken from induction of anaesthesia to loss of eyelash reflex — was observed. Following that, jaw relaxation was assessed as full, partial or difficult and then according to the group either PLMA insertion or ETT (Endotracheal intubation) was performed, without using muscle relaxant.

After pre-insertion preparation and application of water soluble gel on the posterior surface, PLMA was inserted using Index finger insertion technique.

(Technique of PLMA insertion — Hold the PLMA like a pen, with the index finger pushed into the introducer strap. Stabilise the head with the help of the non-dominant hand. Under direct vision, press the tip of the cuff upward against the hard palate and flatten the cuff against it. There should be flexion at the wrist joint. As the index finger passes further into the mouth, the finger joint begins to extend. Advance the device into the hypopharynx until a definite resistance is felt. Full insertion is not possible unless the index finger is fully extended and the wrist is fully flexed. Before removing the finger, the non-dominant hand is brought from behind the patient's head to press down on the airway tube). We did not use the PLMA introducer for the insertion.

Effective airway time (Time between picking up the PLMA / ETT and obtaining the effective airway seal) was also noted in all the cases.[5]

After the placement of PLMA in Group P, the correct position was confirmed by bilateral equal chest movements, normal rectangular shape of the capnograph tracing, bite block to lie between the teeth, absence of gastric insufflation over the epigastrium, no audible leak, the gel displacement test (drainage tube test) and absence of bubbling or movement of the column of lubricant (jelly) placed on the proximal end of the drain tube.[5]

Proper placement of the ETT was checked with bilateral equal air entry and chest rise on ventilation and the normal rectangular shape of the capnograph tracing.

The efficacy of positive pressure ventilation was assessed by adequate chest movement on manual ventilation, bilateral air entry, and normal rectangular shape of the capnograph tracing.

The number of attempts required for the proper placement of PLMA or ETT was recorded. If the first attempt was unsuccessful, a second attempt with repeat induction with Sevoflurane was scheduled in our study. The total number of attempts for PLMA insertion / intubation was recorded in both the groups. The Ryle's tube was inserted in all the patients in both the groups [Table 2].

**Table 2:** Induction characteristics

Parameters	Group P	Group I	*P* value
Induction time (seconds)	205 ± 38.663	214 ± 25.810	> 0.05
Effective airway time (seconds)	31.433 ± 10.358	33.033 ± 4.6199	> 0.05
No. of attempts for PLMA / ETT insertion:			
First attempt	30	30	> 0.05
Second attempt	--	--	
No. of attempts of Ryle's tube insertion:			
First attempt	30	30	> 0.05
Second attempt	--	--	

Anaesthesia was maintained with N_2_O, O_2_ and Sevoflurane in all the cases.

In all the patients, caudal epidural analgesia was given using 0.25% Bupivacaine 2 mg/kg and Tramadol 0.75 mg/kg. After administration of the local anaesthetic drug, the effect of Caudal epidural analgesia was assessed by loss of cremasteric reflex in the male child and abdominal reflex in female child.

Sevoflurane concentration was then gradually reduced to 2% and then to 5% in all the patients, according to the need, and general anaesthesia was maintained throughout the surgical procedure using only Sevoflurane, that is, without using any muscle relaxant. All the patients were manually ventilated throughout the surgical procedure.

Haemodynamic parameters, such as, heart rate, systolic and diastolic blood pressure as well as percentage oxygen saturation and ETCO_2_ were recorded before and during induction and PLMA / ETT insertion, later every 5, 10, 15, 20, 30 minutes during the course of surgery, during removal and in the postoperative period. During the course of surgery, anaesthesia was maintained with O_2_, N_2_O and Sevoflurane and ventilation was adjusted in such a way as to maintain the ETCO_2_ level below 45 mmHg and SPO_2_ above 95%.

After completion of surgery, Sevoflurane and N_2_O were stopped in all patients. They received 100% O_2_ for at least 5 minutes. The haemodynamic parameters were noted down.

PLMA / Tracheal tube were removed when the patients were awakens (facial grimace, adequate tidal volume, ability to open eye). After PLMA removal and tracheal extubation, 100% O_2_ was administered through a face mask for 10 minutes.

Airway-related complications (laryngospasm, bronchospasm, coughing, breath holding and hypoxemia) were recorded. Also secretions over both the ventral and dorsal aspect of the PLMA were checked for any signs of aspiration and regurgitation and pH was checked with the help of litmus paper.

In the postoperative period, incidence of sore throat was recorded and graded as mild, moderate or severe [Table 3] by examining the throat of the patients.

**Table 3:** Analysis of conditions for PLMA insertion and endotracheal intubation

Parameter	Group P	Group I	*'P'* value
Jaw opening			
Full	28 (93.33)	27 (90)	> 0.05
Partial	2 (6.66)	3 (10)	> 0.05
Difficult	--	--	--
Ease of PLMA / ETT insertion	--	--	--
Easy	30 (100)	30 (100)	--
Difficult	00	00	--
Impossible	00	00	--
Coughing	--	--	--
Nil	30 (100)	30 (100)	--
Transient	00	00	--
Persistent	00	00	--
Gagging	--	--	--
Nil	30 (100)	30 (100)	--
Transient	00	00	--
Persistent	00	00	--
Laryngospasm / Airway obstruction	--	--	--
Nil	30 (100)	30 (100)	--
Partial	00	00	--
Total	00	00	--
Patient movements	--	--	--
Nil	30 (100)	28 (93.33)	--
Moderate	00	2 (6.66)	--
Severe	00	00	--

Figures in parentheses are in percentage

All the qualitative data were analysed using the Chi-square test and the quantitative data using student's unpaired t-test. The results were expressed as Mean ± SD. *'P'* value < 0.05 was taken as statistically significant and *'P'* values < 0.001 were taken as highly significant.

## RESULTS

*Demographic data* [Table 1] like age, sex, weight, ASA status were comparable in both the groups. *Mean duration of surgery* (1.5 ± 0.73 hours in Group P and 1.43 ± 0.85 hours in Group I) was also comparable in both the groups.

Males were dominant in both the groups.

As seen in Table 2 *Induction time* (time to loss of eyelash reflex) in Group P (205 ± 38.66 seconds) and in Group I (214 ± 25.81 seconds) were comparable (*P* > 0.05) in both the groups. *Effective airway time* (time between picking up the device and obtaining an effective airway) in Group P (31.43 ± 10.35 seconds) and in Group I (33.03 ± 4.61 seconds) were also comparable (*P* > 0.05) in both the groups. In all the patients, PLMA as well as ETT were inserted at the first *attempt*. (Insertion rate for both PLMA / ETT was 100% in both the groups). *Insertion of Ryle's tube* was done at the first attempt in both the groups. Manipulation was not required in any of the cases.

*Insertion conditions* for PLMA / ETT insertion were comparable (*P* > 0.05) in both the groups as shown in Table 3. In a majority of patients, 93.33% cases in Group P and 90% cases in Group I, we observed full jaw relaxation.

Jaw relaxation was partial in two patients in Group P and three patients in Group I. There was no coughing, laryngospasm or bronchospasm during insertion in both the groups. Two patients in Group I had minimal leg movements during insertion.

In all the patients, caudal epidural analgesia was found to be effective. Supplementation of analgesia was not required in any of the cases in the intraoperative period. We observed that requirement of Sevoflurane was less, that is, 2 to 3% in Group P as compared to 3 to 5% in Group I.

**Figure 2:**
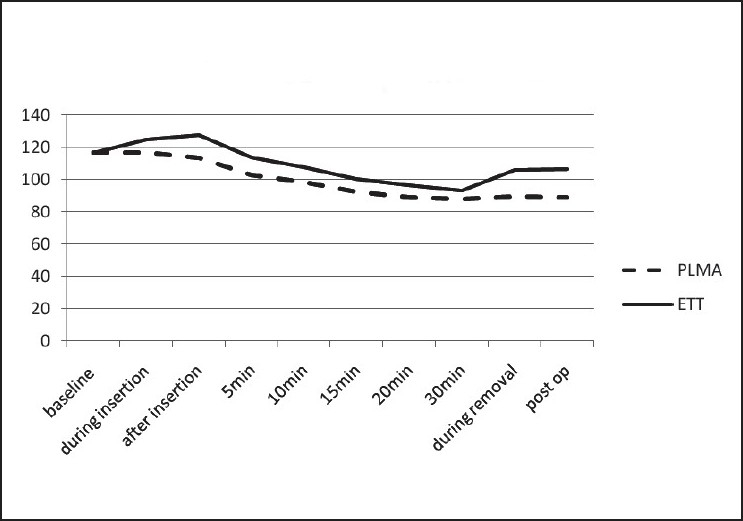
The graph shows changes in heart rate during the procedure. Rise in heart rate was observed in the ETT group as compared to the PLMA group

**Figure 3:**
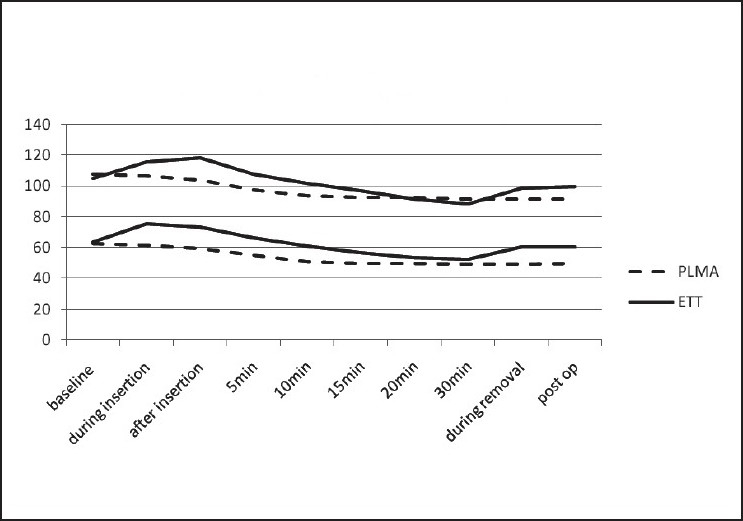
The graph shows changes in systolic (above) and diastolic (below) blood pressure throughout the procedure

There was no change in haemodynamic parameters (heart rate and blood pressure) in Group P during insertion and at the time of removal. In Group I there was a rise in both heart rate and blood pressure during insertion and at the time of extubation, and the change was statistically highly significant. There was a gradual decrease in heart rate and blood pressure in both the groups after induction, at 5-, 10-, 15- and 20-minute intervals [Figures 2 and 3].

There were no significant differences in O_2_ saturation and ETCO_2_ levels within the group as well as between the study groups.

There was one case of misplacement of PLMA after giving lateral position for caudal epidural insertion, but that was corrected immediately and there was no intraoperative complication in that case. Moreover, in the same patient, regurgitation due to displacement of PLMA occurred intraoperatively; the secretions then cleared and manipulation of PLMA was performed and it was repositioned properly. The patient was then managed successfully without any complications.

The pH of secretions on the both the devices was checked using litmus paper in all the cases, after removal. The pH remained within normal limits (7.0 to 7.5) in all cases. In our study, none of the patients had aspiration.

In Group I, 3.33% of the patients experienced postoperative vomiting, 3.33% of the patients had postoperative hypoxemia (SpO_2_ < 90%) (Saturation was immediately maintained > 98% after giving oxygen by face mask), 40% of the patients had coughing and 13.33% had sore throat. Thus, as seen in Table 4, a significant difference was found between Group P and Group I. None of the patients from both the groups had postoperative laryngospasm, bronchospasm, or limb movements at the time of removal of PLMA or extubation. The course of anaesthesia was successful in all the cases.

**Table 4:** Postoperative complications

	Group P	Group I	*'P'* value
Nausea and vomiting	--	1 (3.33)	
Sore throat	--	4 (13.33)	< 0.05
Grade 0 (no)	30	26	
Grade 1 (mild)	--	4	
Grade 2 (severe)	--	--	
Coughing	--	12 (40)	< 0.05
Regurgitation	1 (3.33)	--	< 0.05
Hypoxemia	--	1 (3.33)	< 0.05

Figures in parentheses are in percentage

## DISCUSSION

Paediatric anaesthesia has remained a challenge for anaesthesiologists since the beginning of surgery. It has become an entity by itself and a speciality with the availability of various new airway devices.

Paediatric CLMA has gained wide popularity in paediatric anaesthesia since its introduction into clinical practice. Although at first it was used only as a replacement for the face mask, it is now used as an alternative to ETT.[8]

PLMA is a modified form of classic LMA.[5] The modifications were designed to enable separation of the gastrointestinal tract and respiratory tract, improve the airway seal, enable controlled ventilation and diagnose misplacement.

We decided to compare PLMA with ETT for the safety and efficacy of the airway device in children (Primary goal). We also studied induction time, effective airway time, attempts and conditions during insertion of device, changes in haemodynamics, SpO_2_ and EtCO_2_ as well as airway-related complications (Secondary goal).

**Figure 4:**
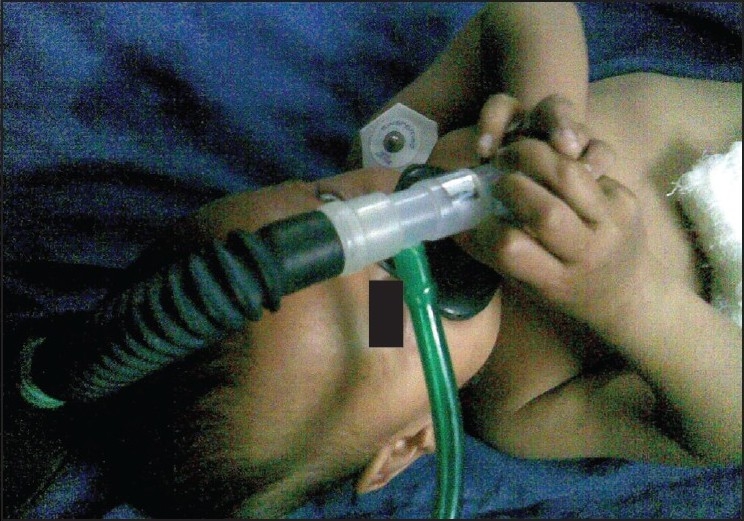
Acceptance of mask and ease of induction with sevoflurane after oral midazolam

Sevoflurane was used as the inhalation agent for induction because, the uptake of the inhalation agent was more rapid in children.[12,13] Furthermore, oral premedication with Midazolam helped us in the smooth and rapid induction of Sevoflurane in the children, as shown in Figure 4.

We used PLMA according to the weight of the child (size 2 for 10 – 20 kg, and 1.5 for 5 – 10 kg) and ETT according to the age of the child for providing ventilation. Both the PLMA and ETT were inserted at the first attempt in all the patients, in other studies first attempt insertion rate was between 90 and 100%.[6,11] In our study there was one case of PLMA misplacement intraoperatively that led to regurgitation, but it was corrected immediately and did not lead to aspiration or fall in saturation.

Insertion conditions, induction time, effective airway time were as shown in Tables 2 and 3 and they were comparable in both groups.

We did not observe any difficulty in carrying out positive pressure ventilation through PLMA.

In our study, as shown in the graphs, the haemodynamic response to PLMA was less as compared to laryngoscopy and intubation, because there was a significant rise in heart rate and blood pressure in the ETT group as compared to the PLMA group, both during insertion and removal of the device. Similar haemodynamic results were also found in other studies.[2,4,6,9,10]

Arterial O_2_ saturation remained constant throughout the study in all the patients and our study was in consonance with other studies. Regarding ETCO_2,_we did not come across any change during the study, while in the study of Nandani *et al*. ETCO_2_ had increased in one patient.

The postoperative complications comprised of mild sore throat (13.3%), coughing (40%) vomiting (3.33%) and hypoxemia (3.33%) in children who were intubated; our study is in consonance with those of the other workers.[1,2] As PLMA was a less invasive airway device, postoperative airway-related complications were less with PLMA than with ETT. In our study we did not come across laryngospasm or bronchospasm in any of the cases; however, Alan Tait *et al*.[11] noted the above complications in their study.

## CONCLUSION

ProSeal^TM^ laryngeal mask airway is a safe and suitable airway device in paediatric patients, as judged by stable haemodynamics, good oxygenation, adequate ventilation and absence of postoperative complications. Hence we can conclude that PLMA can be used as a safe and effective alternative airway device to endotracheal intubation, in children undergoing general anaesthesia.
